# Optimized Cas9:sgRNA delivery efficiently generates biallelic *MSTN* knockout sheep without affecting meat quality

**DOI:** 10.1186/s12864-022-08594-6

**Published:** 2022-05-06

**Authors:** Shiwei Zhou, Peter Kalds, Qi Luo, Kexin Sun, Xiaoe Zhao, Yawei Gao, Bei Cai, Shuhong Huang, Qifang Kou, Bjoern Petersen, Yulin Chen, Baohua Ma, Xiaolong Wang

**Affiliations:** 1 Key Laboratory of Animal Genetics, Breeding and Reproduction of Shaanxi Province, College of Animal Science and Technology, Northwest A&F University, Yangling, China; 2 College of Veterinary Medicine, Northwest A&F University, Yangling, China; 3 Department of Animal and Poultry Production, Faculty of Environmental Agricultural Sciences, Arish University, El-Arish, Egypt; 4 Ningxia Tianyuan Tan Sheep Farm, Hongsibu, China; 5 Institute of Farm Animal Genetics, Friedrich-Loeffler-Institut, Neustadt, Germany

**Keywords:** Genome editing, CRISPR/Cas9 optimization, Homozygous gene knockout, Sheep, *MSTN*, Muscle growth

## Abstract

**Background:**

CRISPR/Cas9-based genome-editing systems have been used to efficiently engineer livestock species with precise genetic alterations intended for biomedical and agricultural applications. Previously, we have successfully generated gene-edited sheep and goats via one-cell-stage embryonic microinjection of a Cas9 mRNA and single-guide RNAs (sgRNAs) mixture. However, most gene-edited animals produced using this approach were heterozygotes. Additionally, non-homozygous gene-editing outcomes may not fully generate the desired phenotype in an efficient manner.

**Results:**

We report the optimization of a Cas9 mRNA-sgRNA delivery system to efficiently generate homozygous *myostatin* (*MSTN*) knockout sheep for improved growth and meat production. Firstly, an sgRNA selection software (sgRNAcas9) was used to preliminarily screen for highly efficient sgRNAs. Ten sgRNAs targeting the *MSTN* gene were selected and validated in vitro using sheep fibroblast cells. Four out of ten sgRNAs (two in exon 1 and two in exon 2) showed a targeting efficiency > 50%. To determine the optimal CRISPR/Cas9 microinjection concentration, four levels of Cas9 mRNA and three levels of sgRNAs in mixtures were injected into sheep embryos. Microinjection of 100 ng/μL Cas9 mRNA and 200 ng/μL sgRNAs resulted in the most improved targeting efficiency. Additionally, using both the highly efficient sgRNAs and the optimal microinjection concentration, *MSTN*-knockout sheep were generated with approximately 50% targeting efficiency, reaching a homozygous knockout efficiency of 25%. Growth rate and meat quality of *MSTN*-edited lambs were also investigated. *MSTN*-knockout lambs exhibited increased body weight and average daily gain. Moreover, pH, drip loss, intramuscular fat, crude protein, and shear force of gluteal muscles of *MSTN*-knockout lambs did not show changes compared to the wild-type lambs.

**Conclusions:**

This study highlights the importance of in vitro evaluation for the optimization of sgRNAs and microinjection dosage of gene editing reagents. This approach enabled efficient engineering of homozygous knockout sheep. Additionally, this study confirms that *MSTN*-knockout lambs does not negatively impact meat quality, thus supporting the adoption of gene editing as tool to improve productivity of farm animals.

**Supplementary Information:**

The online version contains supplementary material available at 10.1186/s12864-022-08594-6.

## Background

Genome editing is a well-established technique for the modification of genomes of living organisms. The application of genome editing in farm animals is promising for agricultural and biomedicine industries [1, 2]. The clustered regularly interspaced short palindromic repeats/CRISPR-associated protein 9 (CRISPR/Cas9) technology [3, 4] has been widely used to induce genome modification, including gene knockout, gene knockin, and single nucleotide substitutions, on a wide range of organisms. CRISPR-based genome editing tools rapidly evolved from the canonical CRISPR/Cas9 system to the more recent CRISPR-associated transposases [5, 6], base editors [7, 8], and prime editors [9, 10]. Although the wide application of CRISPR-based systems, optimization of the delivery methods and parameters of these systems based on target species is still needed. Gene knockout is a favorable approach to disrupt the function of genes that negatively regulate desirable economically important traits in farm animals. However, most gene-edited farm animals generated with zygote microinjection were heterozygous knockouts. Thus, in order to generate authentic homozygous gene-edited animals in a time-saving and cost-effective manner, it is necessary to optimize the gene knockout approach.

At present, the main methods used for genetic modification of animals are somatic cell nuclear transfer (SCNT) of gene-edited cells and embryonic microinjection. SCNT can be used to generate homozygous gene-edited animals, but this approach presents several technical challenges due to high embryonic lethality [11, 12]. On the other hand, microinjection provides a technically less challenging approach for efficient genome modification, being successfully applied to several mammalian species to target crucial genes, such as the *myostatin* (*MSTN*) gene to promote muscle mass gain [13–17]. However, animals with *MSTN* knockout using microinjection usually harbor heterozygous and/or chimeric gene-editing products [18–20].

The *MSTN* gene is a member of the transforming growth factor-beta (TGF-β) superfamily, negatively regulating skeletal muscle tissue production [21–23]. *MSTN* affects the growth and development of muscle tissue by regulating the proliferation of myoblasts [24, 25]. Inactivation of the *MSTN* gene was shown to promote proliferation of myocytes and muscle fiber hypertrophy [26–34]. Therefore, the *MSTN* gene can be considered a genome editing target for exploring the *MSTN* signaling pathway and production of gene-edited animals with improved muscle mass gain and growth rates. Moreover, inducing mutations in both *MSTN* alleles (i.e., in a homozygous manner) may enable a more efficient gene disruption, hence generating animals with more desirable muscle mass phenotype.

Although *MSTN*-knockout sheep models have been generated previously using microinjection of CRISPR/Cas9 reagents [14, 18–20, 35], generation of homozygous *MSTN*-knockout individuals was relatively inefficient, which might be attributed to ineffective sgRNA design and the use of undetermined microinjection concentrations of CRISPR/Cas9 reagents. In most agricultural animal studies, delivery of CRISPR/Cas9 reagents has been performed based on methods established in mice [36]; thus, this approach requires optimization to ensure suitability for agricultural animal studies.

Therefore, in this study, parameters of sgRNAs design and concentration of CRISPR/Cas9 microinjection were optimized. Homozygous *MSTN*-knockout sheep were efficiently generated, and meat quality of *MSTN*-knockout lambs was assessed. This study constitutes a practical reference for generating homozygous knockout farm animals based on the optimization of CRISPR/Cas9 reagents. It was also confirmed that *MSTN* knockout in farm animals increases muscle mass without affecting meat quality.

## Results and discussion

### Design and optimization of sgRNAs in sheep fibroblasts

**Fig. 1:**
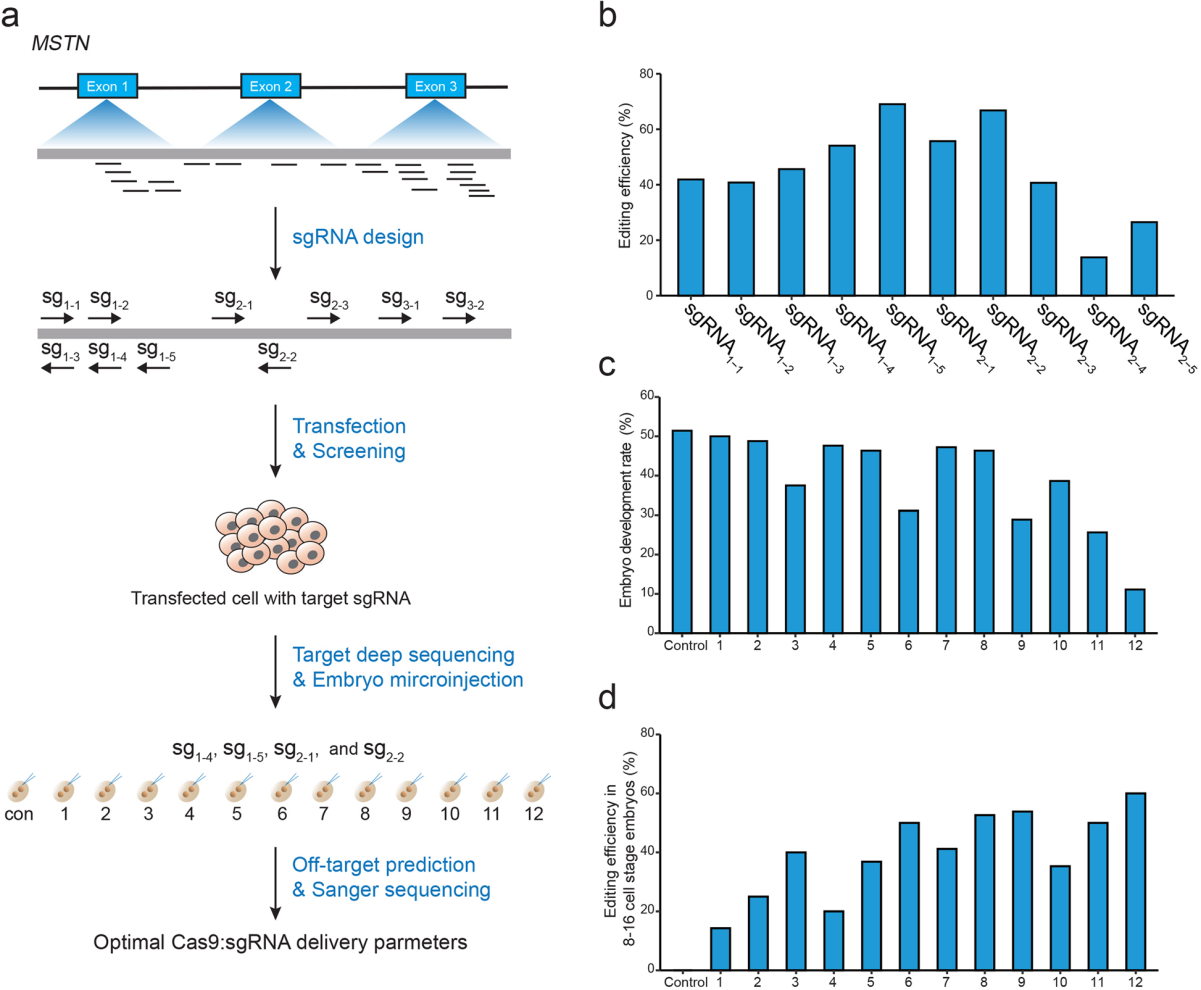
Optimization of the CRISPR/Cas9 system in sheep fibroblasts and embryos. **A** Schematic representation of the study design for the optimization of CRISPR/Cas9:sgRNA delivery system in fibroblasts and sheep microinjected zygotes. **B** Editing efficiency of different sgRNAs targeting the *MSNT* gene in sheep fibroblasts. **C, D** Embryo development rate and editing efficiency for each tested microinjection group. In groups #1, #2, and #3, Cas9 mRNA concentration was 25 ng/μL, whereas concentration of total sgRNAs was 100 ng/μL, 200 ng/μL, and 400 ng/μL, respectively. In groups #4, #5, and #6, Cas9 mRNA concentration was 50 ng/μL, while concentration of total sgRNAs was 100 ng/μL, 200 ng/μL, and 400 ng/μL, respectively. In groups #7, #8, and #9, Cas9 mRNA concentration was 100 ng/μL, while concentration of total sgRNAs was 100 ng/μL, 200 ng/μL, and 400 ng/μL, respectively. In groups #10, #11, and #12, Cas9 mRNA concentration was 400 ng/μL, while concentration of total sgRNAs was 100 ng/μL, 200 ng/μL, and 400 ng/μL, respectively

Within the sheep *MSTN* gene, ten optimal sgRNAs (sgRNA_1–1_, sgRNA_1–2_, sgRNA_1–3_, sgRNA_1–4_, sgRNA_1–5_, sgRNA_2–1_, sgRNA_2–2_, sgRNA_2–3_, sgRNA_3–1_, and sgRNA_3–2_) were selected by sgRNAcas9 and Cas-Offinder software [37, 38]. To evaluate the targeting performance of selected sgRNAs, sheep fetal fibroblasts were co-transfected with plasmids encoding Cas9 and different sgRNAs in six-well culture plates, respectively (Fig. 1a). Forty-eight hours post-transfection, fibroblasts were screened using puromycin and blasticidin for 36 h, and then an antibiotic-free medium was used to enable complete growth of fibroblasts. Genomic DNA was extracted from transfected and drug-screened fibroblasts, and used in PCR amplifications for targeted deep sequencing. Editing efficiency of four sgRNAs (sgRNA_1–4_, sgRNA_1–5_, sgRNA_2–1_, and sgRNA_2–2_) was greater than 50% (Fig. 1b), which is consistent with recent studies [19, 39, 40]. Indeed, the in vitro screening of selected sgRNAs is critical for accurately determining the highly efficient sgRNAs required for downstream experiments at the embryonic and animal levels.

### Determination of optimized microinjection concentration in sheep embryos

Subsequently, the optimal microinjection concentration of CRISPR reagents was determined. Twelve treatment groups (each containing approximately 35–45 embryos) were microinjected with Cas9 mRNA and sgRNAs at different concentrations (see Additional file 1: Table S1). At the 8–16-cell stage, embryos were collected and genomic DNA was amplified, and then target loci were subjected to Sanger sequencing. Notably, higher Cas9/sgRNA concentrations were positively associated with elevated editing efficiency. However, negative correlations with embryo development rates were observed, which is consistent with previous studies [41, 42]. This observation highlights the importance of determining the optimal editing efficiencies and embryo development rates during optimizing the delivery of the CRISPR/Cas9 system [43]. Embryo development rates (8–16-cell stage) of groups #1, #2, #4, #5, #7, and #8 were approximately 50% (Fig. 1c), while editing efficiency of developing embryos of groups #6, #8, #9, and #12 was approximately 50% (Fig. 1d). The overall editing efficiency of group #8 (injection concentration 100 ng/μL Cas9 mRNA and 200 ng/μL sgRNAs) was the highest (24.4%; 10/41) (see Additional file 1: Table S1). Collectively, these results suggest that the concentration of CRISPR components did affect editing performance, and optimal concentration assessment is essential to ensuring high editing efficiency.

### Efficient generation of *MSTN*-homozygous knockout sheep

**Fig. 2:**
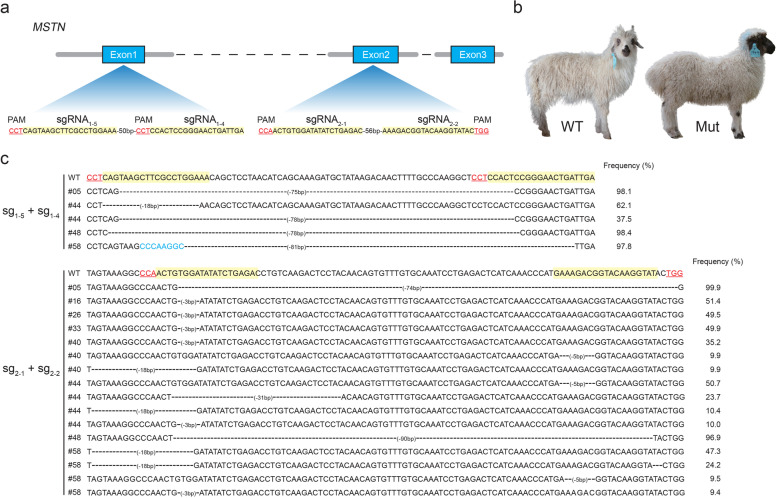
Detection of CRISPR/Cas9-mediated nucleotide variations in founder animals. **A** Schematic diagram of the *MSTN* gene structure and targeting loci of chosen sgRNAs. sgRNAs targeting sites are highlighted in yellow; protospacer adjacent motif (PAM) sequences are highlighted in red. **B** Representative images of a 30-day-old *MSTN* gene-edited (Mut) and wild-type (WT) lambs. **C** Genotypes of target sites in eight founder animals as determined by targeted deep sequencing. Mutations are highlighted in blue and (−) indicates deletions

The ability of the optimized concentrations of CRISPR reagents to generate *MSTN* homozygous knockout sheep with high efficiency was then evaluated. Based on the overall targeting efficiency of treatment group #8, the amounts of 100 ng/μL of Cas9 mRNA and 200 ng/μL of sgRNAs were microinjected into the cytoplasm of one-cell stage embryos. Thirty-three mated female donors were treated for superovulation and subsequently provided 358 one-cell stage fertilized oocytes. Among these, 345 out of 358 microinjected embryos were in adequate condition and were transferred into 58 recipients. Fourteen pregnancies were identified, and 16 lambs (#05, #06, #13, #16, #19, #24, #26, #33, #38, #40, #44, #46, #48, #50, #52, and #58) were born after full-term gestation (approximately 150 days) (Fig. 2b and Table 1).

**Table 1:** Lambs generated with *MSTN* knockout after optimization of the CRISPR/Cas9 system in vitro

No. of donor ewes	33
No. of collected embryos	358
Cas9 mRNA:sgRNA	
No. of microinjected embryos	350
No. of transferred embryos	345
No. of recipient ewes	58
No. of pregnancies	14
Newborns	16
No. of homozygous knockouts	4
No. of non-homozygous knockouts	4
No. of wild-type lambs	8

Genomic DNA was extracted from blood samples of the 16 lambs. Nucleotide sequences around the target loci were amplified by PCR and subjected to Sanger sequencing (see Additional file 1: Table S2). Only exon 2 of the *MSTN* gene was efficiently edited in lambs #16, #26, #33, and #40; both exon 1 and exon 2 were efficiently edited in lambs #05, #44, #48, and #58 (see Additional file 2: Fig. S1). To further define the specific genotypes of gene-edited lambs, PCR amplifications were conducted from genomic DNA obtained from gene-edited lambs #05, #16, #26, #33, #40, #44, #48, and #58 and subjected to targeted deep sequencing. Four lambs (#05, #44, #48, and #58) were homozygous knockouts in exon 1 (Fig. 2c). Taken together, the overall editing efficiency was about 50.0%, which significantly outperforms previous studies using goats (15.3 and 26.5%) [40, 44] and sheep (27.8 and 36.3%) [18, 19]. Thus, these findings confirm the significance of in vitro optimization of sgRNAs and microinjection concentration for higher editing efficiency using CRISPR/Cas9 system.

### Analysis of off-target mutations in gene-edited animals

To evaluate the off-target effects potentially induced by CRISPR/Cas9 system, 10 off-target sites (OT1–OT10) were selected using Cas-OFFinder [38] (see Additional file 1: Table S3). Nucleotide sequences around predicted off-target sites were amplified by PCR from genomic DNA of eight gene-edited founders and evaluated using Sanger sequencing. No off-target editing occurred in gene-edited founders (see Additional file 2: Fig. S2), highlighting the accuracy of the CRISPR/Cas9 system when optimized sgRNAs are used.

### Phenotype assessment of gene-edited animals

**Fig. 3:**
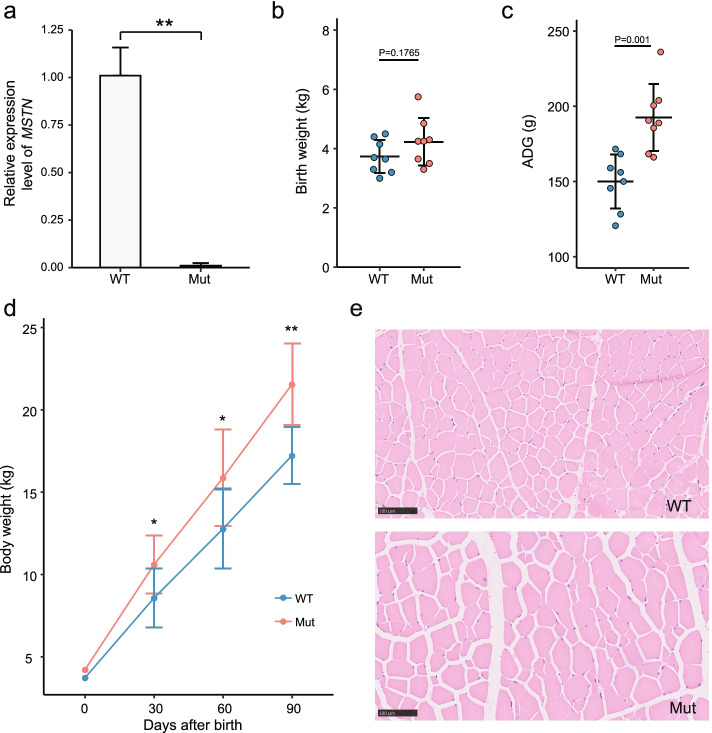
Phenotypic analyses of *MSTN* gene-edited sheep. **A** Expression levels of the *MSTN* gene in homozygous gene-edited (Mut) and wild-type (WT) lambs. **P < 0.01, Student’s *t*-test. **B** Birth weight of Mut and WT lambs; blue dots indicate WT founders and red dots indicate Mut founders. **C** Average daily gain (ADG) of Mut and WT lambs from day 0 to 90. **D** Changes in body weight in Mut and WT lambs from day 0 to 90. *P < 0.05, **P < 0.01, Student’s *t*-test. **E** Histological analysis of muscle tissues of Mut (#48) and a WT founder on day 180

The *MSTN* gene is a negative regulator of muscle growth and development, and loss of *MSTN* causes overdevelopment of muscles in animals, which contributes to the generation of the desirable double-muscling phenotype. To evaluate the expression of *MSTN* in muscle tissues of gene-edited sheep, five lambs [mutated (Mut): #44 and #48; wild-type (WT): #06, #24, and #38] were selected (Fig. 3a). Expression of the *MSTN* gene in gene-knockout lambs was significantly lower than that in WT lambs, which is consistent with previous reports [45, 46].

Next, phenotypes of generated founders were assessed. Average body weight on day (D) 0, 30, 60, and 90 was measured for *MSTN*-knockout lambs (heterozygous and homozygous; *n* = 8) and WT lambs (*n* = 8). Average birth weight of *MSTN*-knockout lambs was higher than that of WT lambs (4.4 kg vs. 3.9 kg, respectively) (Fig. 3b). Moreover, compared to WT lambs, *MSTN*-knockout lambs exhibited remarkably higher body weight on D30, D60, and D90 (Fig. 3d; see Additional file 1: Table S4). Further evaluation of average daily gain from D0 to D90 was conducted; significant differences were observed between *MSTN*-knockout and WT lambs (*P* < 0.01) (Fig. 3c). Collectively, these results confirm that *MSTN*-knockout lambs underwent accelerated postnatal growth.

Then, effects of genetic modification on muscle development of *MSTN*-knockout lambs were assessed using hematoxylin and eosin (H&E) staining. Previous studies highlighted that the loss of *MSTN* function causes an increase in muscle mass, resulting from the combination of hyperplasia and hypertrophy [31, 33, 47]. It is indicated that the increased muscle mass in constitutive *MSTN* knockout mice is primarily due to myofiber hypertrophy [47], while in cattle with a naturally occurring *MSTN* mutation is primarily due to hyperplasia [31, 33]. In the current study, the diameter of myofibers of *MSTN*-knockout lambs (e.g., #48) was notably larger than that of WT lambs (Fig. 3e). These results are consistent with previous gene editing studies conducted on cattle, pigs, goats, and rabbits [17, 40, 48, 49], which showed that disruption of the *MSTN* gene leads to the desirable increased muscle mass phenotype in animals, thus providing a novel way for increased meat production.

### Chemical composition and meat quality analysis of gluteal muscles

Chemical analyses were then performed to assess meat quality parameters of *MSTN*-knockout and WT animals. As shown in Table 2, no differences in pH value, contents of total crude protein, fat, moisture, and shear force of gluteal muscle tissue obtained from *MSTN*-knockout founders and their WT counterparts. Therefore, these results indicate that the editing of the *MSTN* gene in lambs can promote muscle growth without affecting main meat quality parameters and these findings are in line with previous gene editing studies conducted on pigs [50–52]. Additionally, naturally occurring mutations in the sheep *MSTN* gene highlighted similar observations [53–55].

**Table 2:** Effect of *MSTN* knockout on meat quality as assessed on the gluteal muscle tissue of gene-edited and wild-type lambs

Item	Groups		***P*** value
	Control	***MSTN***-edited	
pH_45min_	6.15 ± 0.04	6.14 ± 0.04	0.76
pH_24h_	5.74 ± 0.03	5.74 ± 0.03	1.00
Moisture (%)	78.16 ± 0.48	77.63 ± 0.51	0.13
Drip loss (%)	4.75 ± 0.27	4.63 ± 0.21	0.44
Shear force (N)	46.78 ± 0.77	47.66 ± 0.55	0.063
Intramuscular fat (g/100 g)	4.57 ± 0.49	4.74 ± 0.34	0.55
Crude protein (g/100 g)	18.60 ± 0.35	18.88 ± 0.28	0.21

## Conclusions

In this study, the CRISPR/Cas9 system was optimized for gene editing of *MSTN* in sheep by in vitro selection of highly-efficient sgRNAs. Moreover, the optimal microinjection concentration to efficiently generate biallelic gene knockout animals was determined. Additionally, homozygous *MSTN*-knockout sheep were shown to possess the desirable increased body mass phenotype without affecting meat quality. Therefore, the optimized gene editing system described herein can be potentially applied to enhance desirable traits in food animals.

## Methods

### Animals

All animals were used in the experiments raised at the Ningxia Tianyuan Sheep Farm, Hongsibu, Ningxia Autonomous Region, China. Water and standard feed were supplied ad libitum for both Mut founders and their WT counterparts. Animals were treated according to the Guidelines of Northwest A&F University for the Care and Use of Laboratory Animals, China.

### Screening of highly efficient sgRNAs in sheep fibroblasts

In this study, sgRNAs with NGG as protospacer adjacent motif (PAM) sequence targeting the sheep *MSTN* gene (NCBI gene ID: 443449) were designed using sgRNAcas9 and Cas-OFFinder software packages [37–39]. Ten sgRNAs — five sgRNAs located in the first exon, namely, sgRNA_1–1_, sgRNA_1–2_, sgRNA_1–3_, sgRNA_1–4_, and sgRNA_1–5_; three sgRNAs located in the second exon, namely, sgRNA_2–1_, sgRNA_2–2_, and sgRNA_2–3_; and two sgRNAs located in the third exon, namely, sgRNA_3–1_ and sgRNA_3–2_ — were selected which exhibited predicted high-targeting activity and low off-target efficiency (see Additional file 1: Table S5). The ten groups of sgRNA/Cas9 plasmids were constructed and transfected into cultured sheep fibroblast cells as previously reported [19]. Briefly, sheep fetal fibroblast cells were transfected with 2.5 μg of sgRNA and 5 μg of Cas9 plasmids using Lipofectamine® 3000 Reagent (Invitrogen, Waltham, MA, USA) in a six-well culture plate. After 48 h of transfection, cells were drug-screened with 0.2 μL puromycin (10 μg/μL) and blasticidin (100 μg/μL) added to the medium and maintained for 36 h. Subsequently, the spent medium was replaced with antibiotic-free medium until overgrowth of fibroblast cells was observed. After transfection and drug selection, genomic DNA was extracted and used for targeted deep sequencing. Primers used in amplifications and genotyping of target sites are listed in Additional file 1: Table S2.

### In vitro transcription of sgRNAs and Cas9 mRNA

Oligonucleotides were synthesized and annealed to form double-stranded oligos to construct sgRNAs vectors for in vitro transcription (see Additional file 1: Table S6). Double-stranded oligos were sub-cloned into the pUC57-T7-gRNA vector as previously described [56]. Clones that contained the desired sequences were identified by Sanger sequencing and amplified in culture medium. Plasmids were then obtained using the plasmid extraction kit (AP-MN-P-250G; Axygen, Union City, CA, USA). sgRNAs were in vitro transcribed using the MEGAshortscript Kit (AM1354; Ambion, Austin, TX, USA) and purified using the MEGAClear Kit (AM1908; Ambion, USA). Linearized Cas9 in vitro transcription vector (Addgene; No. 44758) was used as template to produce Cas9 mRNAs as previously described [14].

### Determining the optimal concentration of Cas9 mRNA:sgRNAs at the embryonic level

Four high-efficiency sgRNAs (sgRNA_1–4_, sgRNA_1–5_, sgRNA_2–1_, and sgRNA_2–2_) were selected for further validation experiments in sheep embryos (Fig. 2a). In order to determine the optimum microinjection concentration, 50 healthy ewes (~ 3–5 years old) with normal estrous cycles were selected as donors for zygote collection in October. The procedure of superovulation of donors was carried out as previously described [40]. Briefly, an EAZI-BREED™ controlled internal drug release (CIDR) devise for sheep and goats containing 300 mg of progesterone was inserted into the vagina of donor ewes for 12 days. Superovulation was performed 60 h prior to the removal of the CIDR device using a total of 300 units of FSH (Ningbo Second Hormone Factory, China) in seven injections of 75, 50, 50, 37.5, 37.5, 25, and 25 units at 12 h intervals. A total of 536 zygotes at one-cell stage were collected by surgical operation and immediately placed in TCM-199 medium (Gibco, Waltham, MA, USA). Twelve experimental groups (each containing approximately 35–40 embryos) were microinjected with Cas9 mRNA and sgRNAs at different concentrations. Microinjected embryos were cultured in Quinns Advantage Cleavage Medium (Sage, Newcastle upon Tyne, UK). Embryos were collected at the 8–16-cell stage and frozen at − 80 °C until subsequent analysis. Embryonic genomic DNA was amplified using a Single Cell Whole Genome Amplification Kit (150,343; Qiagen, Hilden, Germany) and used as a template for PCR and Sanger sequencing to determine the rate of editing efficiency (Fig. 1c).

### Generation of *MSTN* homozygous knockout sheep

Embryos were obtained by surgical oviduct flushing from 33 females previously subjected to superovulation. Collected embryos were cytoplasmically coinjected with a mixture of 100 ng/μL of Cas9 mRNA and 200 ng/μL of sgRNAs (as revealed by the optimal result of Group #8 at the embryonic level) using the Eppendorf FemtoJet system. The following parameters were used: microinjection pressure, 45 kPa; compensatory pressure, 7 kPa; and time, 0.1 s. Microinjection was performed in an Olympus ON3 micromanipulation system [39]. Microinjected embryos were cultured in Quinn’s Advantage Cleavage Medium (Sage) for 24 h and subsequently transferred into surrogates as previously reported [19]. Pregnancy was confirmed by observing estrus behaviors in surrogates at each ovulation cycle. After approximately 150 days of pregnancy, 16 newborn lambs were genotyped. Full care and monitoring were given to the lambs after delivery.

### Genotyping of generated founders

Peripheral venous blood samples of two-week-old lambs were collected and submitted to genomic DNA extraction. PCR amplification was conducted using primers listed in Additional file 1: Table S2, and obtained PCR products using the KOD-NEO-Plus enzyme (DR010A; TOYOBO, Japan) were submitted to Sanger sequencing.

### Prediction of off-target sites

Potential off-target sites with maximum three mismatches were predicted using Cas-OFFinder online software [38]. Search for off-target sites was carried out as previously described [38]. Primers used in amplifications for off-target sites and Sanger sequencing are listed in Additional file 1: Table S7.

### Targeted deep sequencing

Target genomic loci were amplified using KAPA HiFi HotStart PCR Kit (KK2501; KAPA Biosystems, Wilmington, MA, USA) for generating a deep sequencing library as previously reported [57]. PCR amplicons were sequenced as a pool using the Dual Index Sequencing with TruSeq HT Library Prep (Illumina, San Diego, CA, USA).

### H&E staining

Samples of gluteus maximus muscle were obtained from *MSTN*-knockout (founder No. #48) and WT (#06) founders (180 days old) for tissue biopsies. The gluteal muscle tissue was immediately fixed with 4% paraformaldehyde at 4 °C overnight, then embedded into paraffin and sectioned. After cutting the samples into 3 μm slices, these slices were stained with H&E. Tissue sections were dewaxed, rehydrated, and stained using standard H&E protocols [19]. After staining, tissue sections were observed by microscopy and images were analyzed.

### Determination of *MSTN* gene expression by real-time PCR

Total RNA was extracted from gluteal muscle tissue of sheep from *MSTN*-knockout (#44 and #48) and control individuals (#06, #24, and #38) using TRIzol™ reagent (Thermo Fisher Scientific, Shanghai, China). PrimeScrip™ RT Reagent Kit with gDNA Eraser (Perfect Real Time; Takara Biomedical Technology, Beijing, China) was used to obtain cDNA. Real-time PCR was performed in ABI Stratagene Mx3000P instrument (Agilent Technologies, Santa Clara, CA, USA) using TB Green Premix Ex Taq II (Takara Biomedical Technology). Primer sequences used in this experiment are listed in Additional file 1: Table S8. Gene expression levels were calculated using the 2^-ΔΔCt^ method and normalized against housekeeping *GAPDH* gene. Each sample was run in triplicate.

### Determination of meat quality

At the age of 6 months, we selected four *MSTN*-edited and four WT female lambs to measure meat quality. Meat quality analysis was performed as previously described [58]. Briefly, pH value was determined after 45 min of gluteal muscle tissue sample collection using a pH-STAT meter (SFK-Technology, Denmark). After 24 h postmortem, gluteal muscle tissue was sampled, as well as pH and shear force of referred samples were determined. Shear force was assessed using the Warner-Bratzler shear force (WBSF) approach [59]. Moreover, crude protein, intramuscular fat, and moisture content of gluteal muscle tissue samples were determined using recognized Association of Official Analytical Chemists (AOAC) methods [58]. Moisture content was determined by drying gluteal muscle tissue samples in an oven at 105 °C until constant weight was obtained. Total crude protein (N × 6.25) was determined using the Kjeldahl method [60]. Intramuscular fat content was determined by the Soxhlet extraction method [61]. Data were reported as g/100 g fresh muscle weight. The data are expressed as the mean ± SEM and analyzed using Student’s *t*-test with significant differences considered at *P* < 0.05.

## Supplementary Information

**Additional file 1: Table S1.** Average cleavage rate and mutation rate of experimental groups. **Table S2.** Primers used for genotyping and amplifying Cas9/sgRNA-targeted *MSTN* fragment. **Table S3.** List of predicted off-target sites. **Table S4.** Growth parameters of *MSTN*-knockout and wild-type sheep. **Table S5.** sgRNA sequences and target sites. **Table S6.** Oligonucleotides used for generating sgRNA-expressing vectors for in vitro transcription. **Table S7.** Primers used for genotyping and amplifying predicted off-target site fragments. **Table S8.** Primers used for measuring *MSTN* expression level by real-time quantitative PCR (RT-qPCR). Description: The file contains the sequences of on- and off-target sites, primers, and oligonucleotides, as well as other relevant data.

**Additional file 2: Figure S1.** Overlapping or discontinuous peaks in Sanger sequencing of DNA samples obtained from the eight founder animals. **Figure S2.** Detection of potential off-targeted sites in the eight founder animals by Sanger sequencing. Ten potential off-targeted sites (OT1-OT10) were predicted by Cas-OFFinder [38]. Description: The file contains the results of Sanger sequencing for on- and off-target loci in gene-edited animals.

## Data Availability

All relevant results are within the article and its Additional files. The raw targeted deep sequencing data are available at NCBI SRA database under BioProject ID: PRJNA785106.

## Abbreviations

sgRNAs: Single-guide RNAs
*MSTN*: *Myostatin*
CRISPR/Cas9: Clustered regularly interspaced short palindromic repeats/CRISPR-associated protein 9
SCNT: Somatic cell nuclear transfer
TGF-β: Transforming growth factor-beta
Mut: Mutant
WT: Wild-type
D: Day
PAM: Protospacer adjacent motif
CIDR: EAZI-BREED™ controlled internal drug release
H&E: Hematoxylin and eosin
WBSF: Warner-Bratzler shear force
AOAC: Association of Official Analytical Chemists
