# Supplementary material for: Optimized Cas9:sgRNA delivery efficiently generates biallelic MSTN knockout sheep without affecting meat quality
Source: BMC Genomics. 2022 May 6;23:348. doi: 10.1186/s12864-022-08594-6 (PMC9078021; doi:10.1186/s12864-022-08594-6)
Supplement: Supplementary file 1 — Additional file 1: Table S1. Average cleavage rate and mutation rate of experimental groups. Table S2. Primers used for genotyping and amplifying Cas9/sgRNA-targeted MSTN fragment. Table S3. List of predicted off-target sites. Table S4. Growth parameters of MSTN-knockout and wild-type sheep. Table S5. sgRNA sequences and target sites. Table S6. Oligonucleotides used for generating sgRNA-expressing vectors for in vitro transcription. Table S7. Primers used for genotyping and amplifying predicted off-target site fragments. Table S8. Primers used for measuring MSTN expression level by real-time quantitative PCR (RT-qPCR). Description: The file contains the sequences of on- and off-target sites, primers, and oligonucleotides, as well as other relevant data. [file 12864_2022_8594_MOESM1_ESM.docx]

**Additional file 1**

**Table S1.** Average cleavage rate and mutation rate of experimental groups.

| **Group** | **Cas9 mRNA**  **(ng.μL^-1^)** | **Total sgRNAs**  **(ng.μL^-1^)** | **No. of microinjected embryos** | **No. of collected 8-16 cell stage embryos (%)** | **Overall targeting**  **efficiency (%)** |
| --- | --- | --- | --- | --- | --- |
| Control | 0 | 0 | 35 | 18 (51.4%) | 0 (0/35) |
| 1 | 25 | 100 | 42 | 21 (50%) | 7.1% (3/42) |
| 2 | 25 | 200 | 41 | 20 (48.7%) | 12.2% (5/41) |
| 3 | 25 | 400 | 40 | 15 (37.5%) | 15.0% (6/40) |
| 4 | 50 | 100 | 42 | 20 (47.6%) | 9.5% (4/42) |
| 5 | 50 | 200 | 41 | 19 (46.3%) | 17.1% (7/41) |
| 6 | 50 | 400 | 45 | 14 (31.1%) | 15.6% (7/45) |
| 7 | 100 | 100 | 36 | 17 (47.2%) | 19.4% (7/36) |
| 8 | 100 | 200 | 41 | 19 (46.3%) | 24.4% (10/41) |
| 9 | 100 | 400 | 45 | 13 (28.9%) | 15.6% (7/45) |
| 10 | 200 | 100 | 44 | 17 (38.6%) | 13.6% (6/44) |
| 11 | 200 | 200 | 39 | 10 (25.6%) | 12.8% (5/39) |
| 12 | 200 | 400 | 45 | 5 (11.1%) | 6.7% (3/45) |

**Table S2.** Primers used for genotyping and amplifying Cas9/sgRNA-targeted *MSTN* fragment.

| **The location of target site** | **Primer** | **Sequence (5'-3')** | **Amplicon (bp)** |
| --- | --- | --- | --- |
| First exon | *MSTN*_1F | GGCCCAGTGGATCTGAATGAG | 436 |
|  | *MSTN*_1R | TCCTCCTTACGTACAAGCCAG |  |
| Second exon | *MSTN*_2F | AGCAGAAGTGCAAGAAAAACCC | 409 |
|  | *MSTN*_2R | ACACATCACAAGGTTTTTAGCATG |  |
| Third exon | *MSTN*_3F | TGCGGTAGGAGAGTGTTTGG | 425 |
|  | *MSTN*_3R | ACAGCGATCTACTACCATGCC |  |

**Table S3.** List of predicted off-target sites.

| Position | 20 | 19 | 18 | 17 | 16 | 15 | 14 | 13 | 12 | 11 | 10 | 9 | 8 | 7 | 6 | 5 | 4 | 3 | 2 | 1 | N | G | G | Location | | | |
| --- | --- | --- | --- | --- | --- | --- | --- | --- | --- | --- | --- | --- | --- | --- | --- | --- | --- | --- | --- | --- | --- | --- | --- | --- | --- | --- | --- |
| sgRNA_1-4_ | T | C | A | A | T | C | A | G | T | T | C | C | C | G | G | A | G | T | G | G | A | G | G | Chr. | Start | End | Strand |
| OT1 | T | C | T | A | T | C | A | G | T | T | C | C | C | G | G | A | G | T | C | A | T | G | G | 11 | 39692699 | 39692721 | – |
| OT2 | T | C | A | G | C | C | A | G | T | T | C | C | C | G | G | C | G | T | G | G | G | G | G | 20 | 13176727 | 13176749 | – |

| Position | 20 | 19 | 18 | 17 | 16 | 15 | 14 | 13 | 12 | 11 | 10 | 9 | 8 | 7 | 6 | 5 | 4 | 3 | 2 | 1 | N | G | G | Location | | | |
| --- | --- | --- | --- | --- | --- | --- | --- | --- | --- | --- | --- | --- | --- | --- | --- | --- | --- | --- | --- | --- | --- | --- | --- | --- | --- | --- | --- |
| sgRNA_1-5_ | T | T | T | C | C | A | G | G | C | G | A | A | G | C | T | T | A | C | T | G | A | G | G | Chr. | Start | End | Strand |
| OT3 | T | T | G | C | C | A | G | G | A | G | A | A | G | C | T | T | A | C | T | G | G | G | G | 4 | 109422548 | 109422570 | – |
| OT4 | T | T | T | C | C | A | A | G | C | C | A | A | G | C | A | T | A | C | T | G | G | G | G | 1 | 141365407 | 141365429 | – |

| Position | 20 | 19 | 18 | 17 | 16 | 15 | 14 | 13 | 12 | 11 | 10 | 9 | 8 | 7 | 6 | 5 | 4 | 3 | 2 | 1 | N | G | G | Location | | | |
| --- | --- | --- | --- | --- | --- | --- | --- | --- | --- | --- | --- | --- | --- | --- | --- | --- | --- | --- | --- | --- | --- | --- | --- | --- | --- | --- | --- |
| sgRNA_2-1_ | A | A | A | G | A | C | G | G | T | A | C | A | A | G | G | T | A | T | A | C | T | G | G | Chr. | Start | End | Strand |
| OT5 | A | A | G | T | A | T | G | G | T | A | C | A | A | G | G | T | A | T | A | C | A | G | G | 6 | 7444201 | 7444223 | + |
| OT6 | A | A | A | G | A | T | G | G | A | A | A | A | A | G | G | T | A | T | A | C | T | G | G | 11 | 58451808 | 58451820 | + |
| OT7 | A | T | A | G | A | C | G | G | T | A | C | A | G | G | T | T | A | T | A | C | A | G | G | 4 | 28547380 | 28547402 | – |

| Position | 20 | 19 | 18 | 17 | 16 | 15 | 14 | 13 | 12 | 11 | 10 | 9 | 8 | 7 | 6 | 5 | 4 | 3 | 2 | 1 | N | G | G | Location | | | |
| --- | --- | --- | --- | --- | --- | --- | --- | --- | --- | --- | --- | --- | --- | --- | --- | --- | --- | --- | --- | --- | --- | --- | --- | --- | --- | --- | --- |
| sgRNA_2-2_ | G | T | C | T | C | A | G | A | T | A | T | A | T | C | C | A | C | A | G | T | T | G | G | Chr. | Start | End | Strand |
| OT8 | G | C | T | T | C | A | G | A | T | A | T | A | T | C | C | C | C | A | G | T | G | G | G | 2 | 159861074 | 159861096 | – |
| OT9 | G | T | C | T | C | T | G | A | T | A | T | A | T | C | T | A | G | A | G | T | A | G | G | 15 | 37302988 | 37303010 | + |
| OT10 | A | C | C | T | C | A | G | A | T | A | T | G | T | C | C | A | C | A | G | T | T | G | G | 19 | 22635442 | 22635464 | + |

**Table S4.** Growth parameters of *MSTN*-knockout and wild-type sheep.

| **Growth parameter** | **Mut**  **(*n*=8; mean)** | **Control**  **(*n*=8; mean)** | ***P* value** |
| --- | --- | --- | --- |
| Birth weight (kg) | 4.23±0.80 | 3.74±0.56 | 0.176 |
| BW at D30 (kg) | 10.61±1.77 | 8.58±1.79 | 0.038 |
| BW at D60 (kg) | 15.88±2.92 | 12.78±2.41 | 0.019 |
| BW at D90 (kg) | 21.56±2.47 | 17.23±1.73 | 0.001 |
| ADG (0-90d) (g) | 192.50±22.17 | 149.93±18.04 | 0.001 |

BW, Body weight; ADG, Average daily weight gain. Eight animals were used for Student's *t*-test.

**Table S5.** sgRNA sequences and target sites.

| **sgRNA** | **Targeting site** | **Location** | **Strand** |
| --- | --- | --- | --- |
| *MSTN*-sgRNA_1-1_ | CTACCACGTTACGACGGAAACGG | Chr2:129060084-129060106 | + |
| *MSTN*-sgRNA_1-2_ | CGATGACTACCACGTTACGACGG | Chr2:129060078-129060100 | + |
| *MSTN*-sgRNA_1-3_ | CGTACTGATCAATCAGTTCCCGG | Chr2:129060016-129060038 | - |
| *MSTN*-sgRNA_1-4_ | TCAATCAGTTCCCGGAGTGGAGG | Chr2:129060007-129060129 | - |
| *MSTN*-sgRNA_1-5_ | TTTCCAGGCGAAGCTTACTGAGG | Chr2:129059934-129059956 | - |
| *MSTN*-sgRNA_2-1_ | GTCTCAGATATATCCACAGTTGG | Chr2:129062828-129062850 | + |
| *MSTN*-sgRNA_2-2_ | CCAGTATACCTTGTACCGTCTTT | Chr2:129062049-129062071 | - |
| *MSTN*-sgRNA_2-3_ | CCCATGAAAGACGGTACAAGGTA | Chr2:129062122-129062144 | - |
| *MSTN*-sgRNA_3-1_ | TATAAGGCCAATTACTGCTCTGG | Chr2:129064527-129064549 | + |
| *MSTN*-sgRNA_3-2_ | GGATTTTGAAGCTTTTGGATGGG | Chr2:129064481-129064503 | + |

**Table S6.** Oligonucleotides used for generating sgRNA-expressing vectors for *in vitro* transcription.

| **Primer** | **Sequence (5'-3')** |
| --- | --- |
| *MSTN*_sgRNA_1-4_ Top strand | TAGGTCAATCAGTTCCCGGAGTGG |
| *MSTN*_sgRNA_1-4_ Bottom strand | AAACCAGTAAGCTTCGCCTGGAAA |
| *MSTN*_sgRNA_1-5_ Top strand | TAGGTTTCCAGGCGAAGCTTACTG |
| *MSTN*_sgRNA_1-5_ Bottom strand | AAACCAGTAAGCTTCGCCTGGAAA |
| *MSTN*_sgRNA_2-1_ Top strand | TAGGAAAGACGGTACAAGGTATAC |
| *MSTN*_sgRNA_2-1_ Bottom strand | AAACGTATACCTTGTACCGTCTTT |
| *MSTN*_sgRNA_2-2_ Top strand | TAGGTCTCAGATATATCCACAGT |
| *MSTN*_sgRNA_2-2_ Bottom strand | AAACACTGTGGATATATCTGAGA |

**Table S7.** Primers used for genotyping and amplifying predicted off-target site fragments.

| **Off-target site** | **Sequence (5'-3')** | **Amplicon (bp)** |
| --- | --- | --- |
| OT1_F | GTGCTGGGTCTGAACTGACT | 578 |
| OT1_R | CAGCTTTCTCAGCTCCGAGT |  |
| OT2_F | TCACCTGCCCCAAGGAATTG | 430 |
| OT2_R | ATGAGGGAACGGTAACAGGC |  |
| OT3_F | CCGCATTTGGGGGACATAGT | 417 |
| OT3_R | GATGGTTCTGGGGCTGTGAA |  |
| OT4_F | CCTCAGTAGGATTGAAGGGGC | 605 |
| OT4_R | GGGGTTCATGTTTGGGAACG |  |
| OT5_F | ACAACATGGGCTGGAAGTGTT | 633 |
| OT5_R | TCATTGTTGGGCTCCTCTTCC |  |
| OT6_F  OT6_R | GACAGACAAATAAACGGAT | 1099 |
|  | ATATGTATAACAAATTCGCTT |  |
| OT7_F | CAGCACCTAGCACAGTTCCT | 659 |
| OT7_R | CCAGGGGGTCAGTGAATGAG |  |
| OT8_F | GGTACCCTGGTTGTTCACACG | 585 |
| OT8_R | AGGAGGAGAAGGGGAAGTCCA |  |
| OT9_F | ATGTGCTCATGCTCTGCTCA | 553 |
| OT9_R | CATGCTCTTAGCCAGCACAC |  |
| OT10_F | GGAGTGGAAAAGAGGAGCTGG | 532 |
| OT10_R | CATGGACTAGGCCAGTGGTCA |  |

**Table S8.** Primers used for measuring *MSTN* expression level by real-time quantitative PCR (RT-qPCR).

| **Primer** | **Sequence (5'-3')** |
| --- | --- |
| *MSTN*_F | CTTTTGCCCAAGGCTCCTCCA |
| *MSTN*_R | ACATTTGGGTTTTTCTTGCACT |
