# Supplementary material for: Optimized Cas9:sgRNA delivery efficiently generates biallelic MSTN knockout sheep without affecting meat quality
Source: BMC Genomics. 2022 May 6;23:348. doi: 10.1186/s12864-022-08594-6 (PMC9078021; doi:10.1186/s12864-022-08594-6)
Supplement: Supplementary file 2 — Additional file 2: Figure S1. Overlapping or discontinuous peaks in Sanger sequencing of DNA samples obtained from the eight founder animals. Figure S2. Detection of potential off-targeted sites in the eight founder animals by Sanger sequencing. Ten potential off-targeted sites (OT1-OT10) were predicted by Cas-OFFinder [38]. Description: The file contains the results of Sanger sequencing for on- and off-target loci in gene-edited animals. [file 12864_2022_8594_MOESM2_ESM.docx]

**Additional file 2**

**
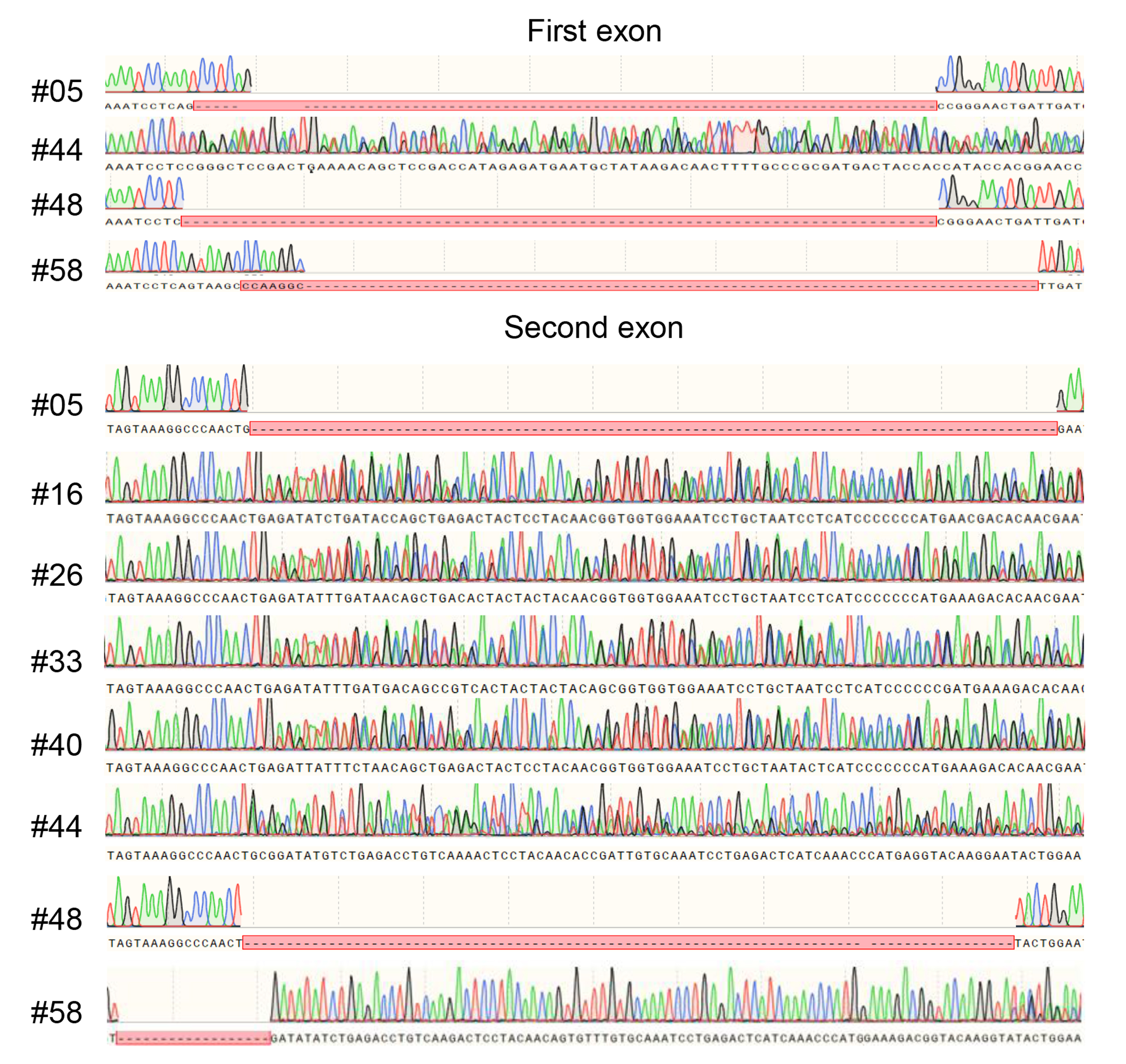
**

**Figure S1.** Overlapping or discontinuous peaks in Sanger sequencing of DNA samples obtained from the eight founder animals.

**
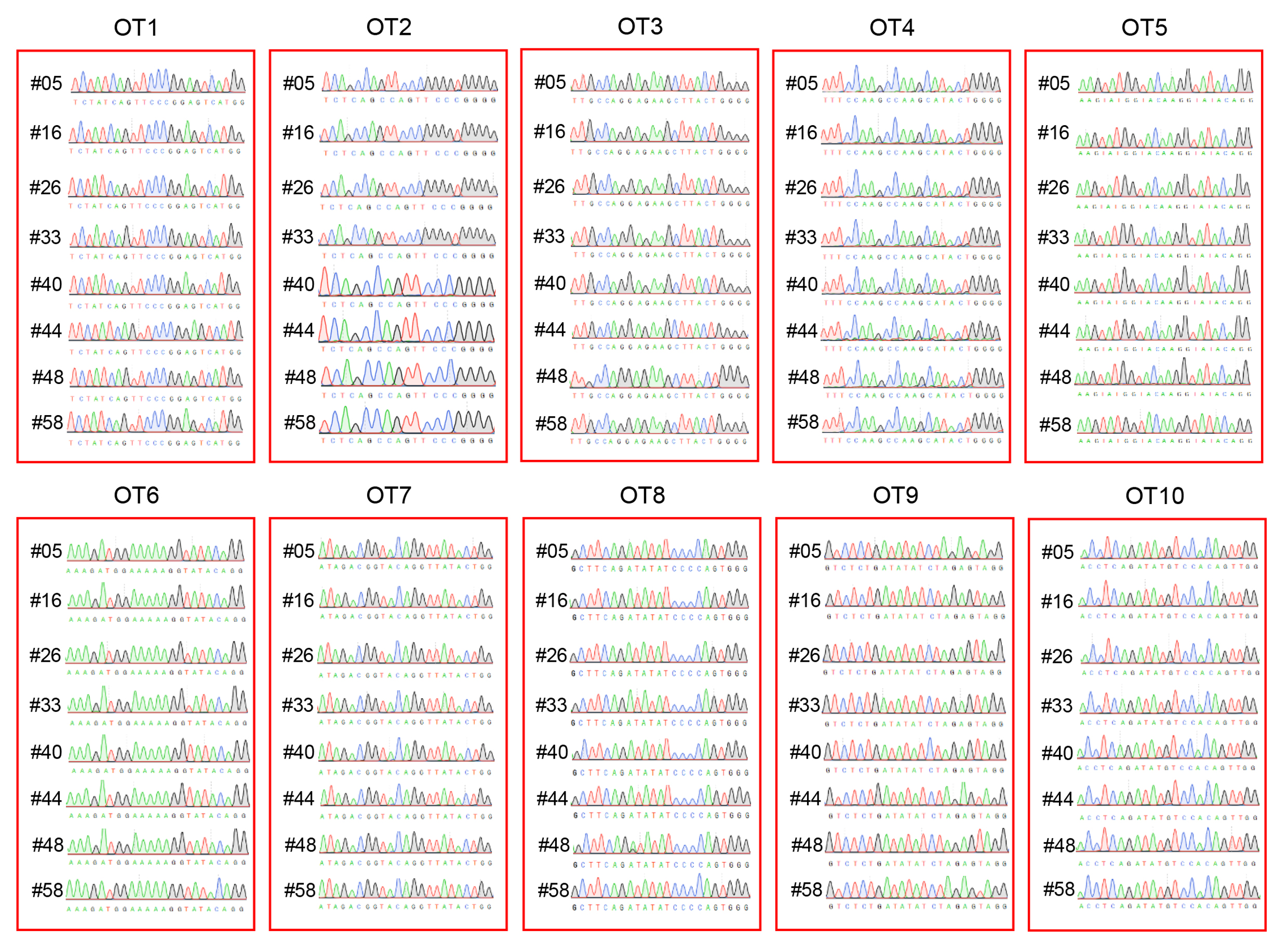
**

**Figure S2.** Detection of potential off-targeted sites in the eight founder animals by Sanger sequencing. Ten potential off-targeted sites (OT1-OT10) were predicted by Cas-OFFinder [38].
